# A Systematic Review of Commercial Cognitive Training Devices: Implications for Use in Sport

**DOI:** 10.3389/fpsyg.2018.00709

**Published:** 2018-05-11

**Authors:** David J. Harris, Mark R. Wilson, Samuel J. Vine

**Affiliations:** Sport and Health Sciences, University of Exeter, Exeter, United Kingdom

**Keywords:** cognitive training, brain training, attention, sport, working memory, sport performance

## Abstract

**Background:** Cognitive training (CT) aims to develop a range of skills, like attention and decision-making, through targeted training of core cognitive functions. While CT can target context specific skills, like movement anticipation, much CT is domain general, focusing on core abilities (e.g., selective attention) for transfer to a range of real-world tasks, such as spotting opponents. Commercial CT (CCT) devices are highly appealing for athletes and coaches due to their ease of use and eye-catching marketing claims. The extent to which this training transfers to performance in the sporting arena is, however, unclear. Therefore, this paper sought to provide a systematic review of evidence for beneficial training effects of CCT devices and evaluate their application to sport.

**Methods:** An extensive search of electronic databases (PubMed, PsychInfo, GoogleScholar, and SportDiscus) was conducted to identify peer-reviewed evidence of training interventions with commercially available CT devices. Forty-three studies met the inclusion criteria and were retained for quality assessment and synthesis of results. Seventeen studies assessed transfer effects beyond laboratory cognitive tests, but only 1 directly assessed transfer to a sporting task.

**Results:** The review of evidence showed limited support for far transfer benefits from CCT devices to sporting tasks, mainly because studies did not target the sporting environment. Additionally, a number of methodological issues with the CCT literature were identified, including small sample sizes, lack of retention tests, and limited replication of findings by researchers independent of the commercial product. Therefore, evidence for sporting benefits is currently limited by the paucity of representative transfer tests and a focus on populations with health conditions.

**Conclusions:** Currently there is little direct evidence that the use of CCT devices can transfer to benefits for sporting performance. This conclusion, however, stems more from a lack of experimental studies in the sporting field and a lack of experimental rigor, rather than convincing null effects. Subsequently, there is an opportunity for researchers to develop more reliable findings in this area through systematic assessment in athletic populations and major methodological improvements.

## Introduction

### Rationale

Over the past 10 years, cognitive training (also known as brain training, perceptual training, attention training, or mind training) has boomed, both as a research topic, and as a commercial product. The overall cognitive training (CT) and assessment market is currently worth $1.98 billion (US) and set to rise to over $8 billion by 2021 (marketsandmarkets.com, 2017). Currently, however, it is unclear to what extent device popularity and marketing claims align with scientific evidence. While many commercial CT (CCT) programmes are based on well researched cognitive tasks that have shown trainability (Shipstead et al., 2012; Harrison et al., 2013; Melby-Lervåg and Hulme, 2013), marketing claims suggest more extensive benefits for boosting general brain power and aiding daily mental function (Simons et al., 2016). Additionally, companies cite scientific evidence for their products, which often relates to the basic cognitive tasks rather than direct testing of their device.

CCT devices that allow the user to download an application or log on to a website and immediately begin training can be referred to as “off-the-shelf” devices. They require no instruction or expertise to use, and can often be run on just a mobile phone or computer. Such devices are highly appealing for sport, as they claim to enhance a range of skills, such as attention, speed of processing, decision-making and problem solving, and can be practiced at the athlete's convenience. Given the recent proliferation of these devices and controversy in the academic literature regarding their efficacy, we aim to provide a systematic appraisal of the peer-reviewed evidence for CCT devices. As thesedevices hold particular interest for developing the cognitive skills of athletes, we will also evaluate the evidence for transfer to the sporting domain.

CT consists of systematic practice on tasks intended to develop abilities such as working memory and attention, for transfer to other tasks and settings (Simons et al., 2016). Domain-general CT, which seeks to develop core functions applicable to a multitude of tasks, can be distinguished from context-specific CT such as training perceptual-cognitive abilities using the expert performance approach (Ericsson, 2003), which targets cognitive skills in a specific task (e.g., tennis serve anticipation). Further, the aforementioned commercial devices are distinct from the, often bespoke, methods used exclusively for research (e.g., Jaeggi et al., 2011; Ducrocq et al., 2016, 2017). Here, we are primarily concerned with commercially available methods that aim to enhance domain general abilities. The scientific rationale for CT largely stems from the concept of “neuroplasticity,” which claims that the brain, much like a muscle, can change and adapt to challenges, and that targeted conditioning of a specific region will cause a sustained development in size and/or functional capacity (Draganski et al., 2004). Such adaptation, evident in both young and old (Mahncke et al., 2006; Schlaug et al., 2009), could facilitate a wide range of benefits that are supposedly harnessed through CT, including memory, attention, processing speed, fluid intelligence, problem-solving, and learning abilities (Simons et al., 2016) (see Table 1 for descriptions of cognitive functions). The end goal of CT is to achieve (1) improvements in the cognitive function that was trained (near transfer); (2) improvements in other associated or “overlapping” cognitive functions (e.g., after training working memory, are improvements in attentional control achieved?); and finally, (3) improvements in the performance of tasks in the real world that utilize those cognitive functions (far transfer) (Simons et al., 2016). As such, context general CT relies heavily on the proposition of domain generality; that is, the belief that training-related improvements in domain-specific abilities will transfer onto more general cognitions and skills (Baddeley, 1986; Dahlin et al., 2008).

**Table 1:** Description of cognitive functions targeted by CT training devices included in the systematic review.

**Cognitive function**	**Description *(and tests)***
Working memory (WM)	A limited cognitive capacity that is responsible for temporarily holding information for active manipulation. Consists of visuospatial and phonological components, which are supervised by a central executive. WM underpins any functions that require storage and use of information*. Digit, letter, and spatial span tasks that require information to be held during a simultaneous mental load (e.g., tone counting), also N-back, Operation Span Task*.
Executive function (EF)	A multi-component construct that consists of a range of processes involved in the planning, organization, coordination, implementation, and evaluation of many non-routine activities. Plays a key role in allocating attention and higher-level functions. *Wisconsin Card Sorting Test, verbal fluency test*.
Inhibition	A sub-function of WM and aspect of executive function which actively suppresses irrelevant or unwanted information. *Stroop test, Posner Flanker task, Go/NoGo*.
Shifting/Switching	An aspect of executive function responsible for switching between multiple tasks. May be a function of WM. *Wisconsin Card Sorting Test, Trail Making Test*.
Divided attention	The ability to attend to and process two tasks or sources of information at the same time, e.g., two spatial locations. Requires shifting function. *Multiple object tracking, dual-task paradigms*.
Selective attention	The ability to attend to some stimuli while disregarding others that are irrelevant to the task at hand, for example, an individual's ability to search for a single letter among an array of distracting and irrelevant letters. Requires inhibition function. *Visual search, dichotic listening*.
Sustained attention	One's ability to maintain a focus of attention on one task for a sustained period of time. *Sustained Attention to Response Task*.
Fluid intelligence	The domain general ability to solve new problems and reason. *Wechsler Adult Intelligence Scale, Raven's Progressive Matrices*.
Crystalline intelligence	The ability to use learned knowledge and experience. *Sentence completion, verbal classification*.
Processing speed	Time taken to take in, process and respond to information. Can be domain specific, e.g., visual or verbal. *Useful field of view, reaction times, Paced Auditory Serial Addition Test*.
Short term memory (STM)	The temporary, limited capacity, passive store that holds information to be used in WM. Also referred to as episodic memory. *Span tasks, Corsi Block Test*.
Reasoning	The process of making judgments or conclusions based on logical processing. Very similar to fluid intelligence. *Tower of London, Tower of Hanoi*.

In order to evaluate the efficacy of current commercially available devices, it is necessary to outline the criteria through which existing research will be appraised. In order to determine causal effects, only studies in which training interventions are used will be considered. Of these, randomized, double-blind clinical trials provide the gold standard. A recent review of CT by Simons et al. (2016) outlines five key questions for assessing the evidence for a training device:

1. Has the training demonstrated transfer of training to other laboratory tasks that measure the same cognitive function as the training?
2. Has the training demonstrated transfer to relevant real-world tasks?
3. Has the training been evaluated using an active control group whose members have the same expectations of training benefits as the members of the experimental group?
4. How long are the trained skills retained?
5. Have the purported benefits of the training been replicated by research groups other than those selling the product?

These questions will be central to our assessment of the current literature on commercial devices. Firstly, the device must demonstrate robust evidence that it does indeed enhance the cognitive function it purports to train, through near transfer to similar tests. If not, subsequent considerations are immaterial. Secondly, and crucially for applications to sport, it must show evidence of transfer to real-world tasks. Thirdly, good experimental design requires the use of *active* control groups where participants expect a training benefit. Simons and colleagues identify the poor use of control groups in much CT research, where the use of passive controls means that training effects may be due to the expectations of the training group. Fourthly, if CT makes use of “neuroplasticity,” changes in cognitive function in response to training can lead to long term neural changes, which should be retained over time (Park and Bischof, 2013). Finally, much research on commercial devices has been conducted by researchers linked to the companies selling the products. Therefore, in order for research to be considered reliable, the findings should be replicable by researchers independent of the company. These critical questions will be used to identify the strength of evidence for each training device.

CT is typically adopted in the following contexts: (1) compensatory—to overcome or circumnavigate cognitive deficits (Rapport et al., 2013); (2) restorative—to rediscover or restore lost cognitive functions; or (3) additive—to enhance or build upon existing cognitive functions (Ward et al., 2008). Benefits for sport fall into the third context. Currently, however, commercial devices have received little direct testing in athletes or other healthy populations, but considerable testing in older adults and populations with health conditions, where the device aims to overcome deficits in cognitive function. As such, most of the existing findings relate to compensatory or restorative rather than additive ergogenic effects. These findings remain imperative for evaluating the general effectiveness of CT devices, but generalizing to athletes is more difficult. Therefore, reviewed studies will be divided based on the use of young and healthy (additive) versus aged and non-healthy (compensatory/restorative) samples. In doing this, we aim to answer two questions; (1) Is there reliable evidence for any far transfer benefits (all adult populations), following training with CCT devices? (2) Is there reliable evidence for transfer to sporting tasks, following training with CCT devices?

Performing optimally in sport requires a range of cognitive skills, like selective attention (Abernethy, 1987), divided attention (Memmert, 2009) and working memory (Furley and Memmert, 2010), particularly when under pressure (Eysenck and Wilson, 2016). Recent findings suggest that training these functions may transfer to sport, as Ducrocq et al. (2016) demonstrated that training on a bespoke attentional task targeting the inhibition function of working memory improved pressurized volley performance in recreational tennis players. Perceptual-cognitive training, a form of CT that aims to train perceptual and sensory functions responsible for decision-making and anticipatory skills, has also shown cognitive benefits. Typically, life-sized video is used to replicate key situations from the performance environment, enabling trainees to develop the cognitive functions that are utilized in the real world (Williams et al., 2002). This approach has demonstrated benefits for skills like anticipation (see Broadbent et al., 2015 for review). Alternatively, vision training, such as Quiet Eye Training, uses videos of eye movements to teach expert-like gaze strategies to novices. This approach has shown substantial benefits in perceptual-motor as well as perceptual-cognitive tasks (see Vine et al., 2014 for review). Consequently, there is robust evidence for enhancing sporting performance through other methods of cognitive enhancement. The fundamental question is whether these benefits can also be achieved by CCT devices that purport to train domain general abilities (as Jaeggi et al., 2011; Ducrocq et al., 2016), rather than task specific perceptual or attentional abilities?

### Research question

CT can take several forms, based on the purpose of the device and method of training. In particular, commercial devices, like smart phone based braining training games, can be distinguished from non-commercial devices, such as bespoke methods for research (e.g., Jaeggi et al., 2011; Ducrocq et al., 2016, 2017). Additionally, CT can be either truly domain general, or context-specific, such as training of sport specific perceptual-cognitive abilities (Broadbent et al., 2015) and task-specific visual training (Vine et al., 2014). While these methods hold promise for sport, they are highly specialized and often require expert instruction, limiting potential for general usage. Therefore, we aim to review devices that are commercially available for use by a range of sportspeople, and target domain-general skills. CCT devices have the potential to provide an affordable and convenient way of regularly training cognitive skills. This ease of use, in combination with the far-reaching marketing claims, means that CCT devices can be attractive to coaches and athletes. It is currently unclear, however, if these devices can provide reliable transfer to sporting skills. Therefore, we aim to systematically review existing evidence for the use of these devices. Specifically, we firstly assess evidence for performance enhancement across a range of adult populations, and secondly evidence for potential benefits in the sporting arena. We also aim to evaluate study quality to inform future research in this area.

## Methods

### Search strategy

The methodology employed for the systematic review was based on the guidelines described by Khan et al. (2003). The aim of the review was to summarize and synthesize peer-reviewed research relating to the effectiveness of CCT devices in adult populations, firstly relating to compensatory/restorative^1^ effects, and secondly with regards to potential transfer to sport. Only devices claiming to directly train domain general cognitive function were reviewed. For instance, there is evidence for the beneficial effects of exercise and mindfulness training for cognitive function (Cassilhas et al., 2007; Howells et al., 2016), but our search was restricted to devices specifically designed for CCT. Additionally the search was restricted to studies investigating performance enhancement in adult populations. To this end, an electronic search of PubMed, PsycInfo, GoogleScholar, and SPORTDiscus databases was conducted, for research relating to CCT devices, up to, and including, September 2017. The initial search was performed in PubMed and adapted to the other databases. Key search terms were *cognitive, brain, working memory*, or *attention*, combined with *training*, and excluded titles containing *children*. Research sections of websites for CCT devices identified in the database search were an additional source of papers. These included the websites for Neurotracker, Cogmed, Cognifit, Lumosity, Posit Science, and Dynavision. Further studies were identified through searching reference lists. The retrieved results were initially assessed for relevance based on their title and abstract, with studies that were ineligible, irrelevant, or duplicates removed. Next the remaining results were screened based on the full-text article, with further ineligible or irrelevant results removed.

### Selection of studies

Included studies were required to meet the following criteria: (1) test a commercially available device, (2) be in a peer-reviewed, English language journal, (3) use adult participants (18+ years of age), (4) use a training intervention (i.e., assigned groups to device practice for any time duration), (5) assess either near or far transfer^2^, and (6) accurately represent the commercial device (i.e., when a device employs multiple subtasks, all tasks were used and training groups did not use more than one CT device).

**Figure 1:**
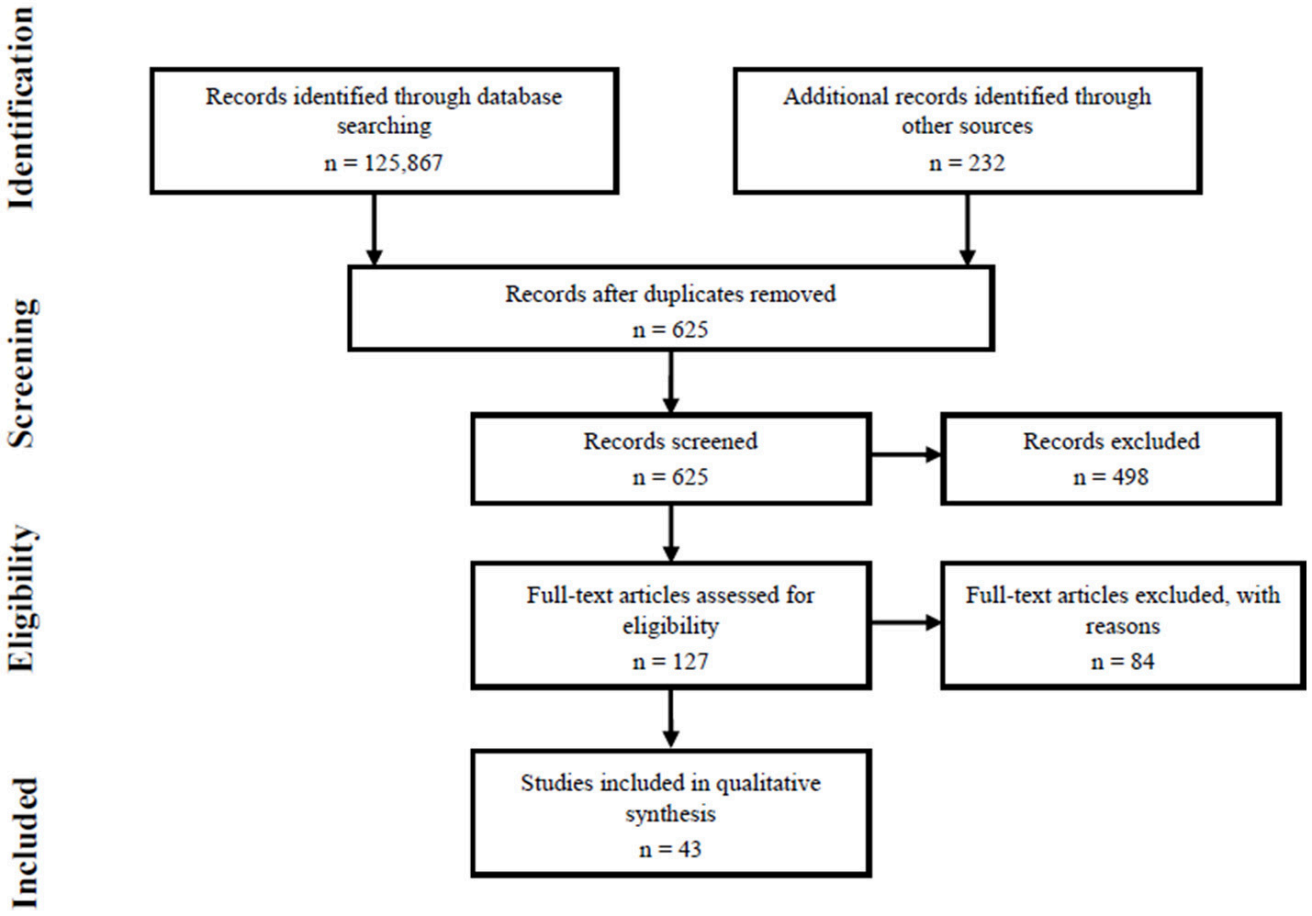
Four-phase PRISMA flow diagram.

The identification and selection of papers was guided by the four-phase flow diagram of the Preferred Reporting Items for Systematic Reviews and Meta-Analyses (PRISMA: Figure 1).

### Data extraction and quality assessment

After all articles fitting the search criteria were obtained, they were assessed for quality and key data was extracted for the summary table (**Tables 3A**,**B**). Data extraction summarized the following information from each paper: authors; participant population; name of the training device; was an active control group used (if yes, what); was a near transfer test included (yes/no); was a far transfer test included (yes/no); was a retention test used (yes/no); were researchers independent of the company marketing the device (yes/no); which cognitive functions were assessed (Table 1 for descriptions); summary of findings. For consistency, discussion and crosschecking of included studies was carried out amongst the authors.

Study quality was determined by evaluating the internal and external validity of the selected studies. Items for assessing study quality (Appendix 2 in Supplementary Material) were taken from the Quality Index (Downs and Black, 1998), the Epidemiological Appraisal Instrument (Genaidy et al., 2007) and Durant's (1994) checklist for the evaluation of research articles. The five critical questions relating specifically to CT research taken from Simons et al. (2016) review were also included. This formed a 22 item checklist that was scored 1 when a criterion was met and 0 when it was not (or was unknown). This gives a maximum score of 22 for the highest possible quality. The quality assessment was primarily conducted by one author, with queries discussed among the remaining authors (Appendix 1 in Supplementary Material).

## Results

### Search results

The initial database searches returned 125,867 papers which, after screening for relevance and matching to inclusion criteria (Figure 1), resulted in 43 papers to be reviewed against the quality assessment criteria (Appendix 2 in Supplementary Material).

### Characteristics of included studies

The included studies resulted in seven devices for review, these were (with number of studies); Cogmed (15), Lumosity (9), Insight and Brain Fitness by Posit Science (6), Cognifit (4), Neurotracker (4), Nintendo Big Brain Academy and Brain Age (4), and Dynavision (1). The participant samples included populations that were healthy and those with health conditions, 27 with participants from healthy young (<60 years) or old (>60 years) adult groups, four focusing on ADHD, nine on brain injury and cognitive impairments, two on cancer survivors and one on participants living with depression. There was one study with participants from the armed forces and only one in a sporting population.

Twenty-one studies assessed far transfer^3^, that is, to a measure other than a cognitive test, such as driving ability or soccer passing. Fourteen of these, however, were self-report measures such as quality of life, perceived cognitive function and health condition symptoms. The non-self-report transfer measures were expert ratings of motor skill and safety to drive, ability to perceive human motion, sleep quality, soccer passing ability, and two direct neural measures. Only two studies did not assess near transfer, both of which were studies assessing Neurotracker focusing on far transfer.

### Summary of evidence for CT devices

An overview of each CCT device is provided in Table 2, and a summary of findings from each study is included in Table 3A (compensatory/restorative effects) and Table 3B (additive effects). Here we give an overview of the evidence for each device, in relation to the five critical questions.

**Table 2:** Summary of devices identified in the systematic review.

**Device**	**Target participants**	**Device description**	**Cognitive function(s) trained**	**Delivery of training**
Cogmed (www.cogmed.com/)	Children and adults with memory and attention problems.	A market leader, Cogmed has been adopted by numerous intervention studies for memory and attention impairments (e.g., Brehmer et al., 2012; Åkerlund et al., 2013; Dunning et al., 2013).	WM capacity, general attentional abilities.	Mobile and computer application. Tasks are typical of traditional cognitive tests, such as digit and letter span and focus heavily on working memory. The tasks are described as “adaptive,” as they become progressively harder as users improve.
Lumosity (www.lumosity.com/)	General population and those with memory deficits.	One of the leading sources for online brain training, providing over 40 brain training games.	Speed of processing, memory, attention, flexibility, and problem-solving.	Website and Mobile application. Uses a range of games based on cognitive tests, focusing on speed of processing.
Posit Science (www.brainhq.com/)	General population.	Two brain-training products from Posit Science were identified in the review, Brain Fitness and InSight, which are now part of the BrainHQ programme.	Speed of processing, WM.	The speed of processing games used by Posit Science are based on a useful field of view task used in a large clinical trial (the ACTIVE trial, Ball et al., 2002).
Cognifit (www.cognifit.com/)	General population, and those with declining function.	Initially focused on cognitive training for driving performance, Cognifit claims to measure, train, and properly monitor various applied cognitive skills and their relation to neurological pathologies.	Numerous cognitive skills including working memory, divided attention, and processing speed	Online and mobile application. Visual, auditory, and cross-modal tasks including puzzles, problem solving, and reaction time games.
NeuroTracker (www.neurotracker.net/)	Athletes and military.	Used by elite teams in sports such as Soccer and American Football. Training is based upon 3D multiple object tracking (Pylyshyn and Storm, 1988) which requires processing of dynamic stimuli.	Attention, WM, and visual information processing speed.	The user tracks target 3D balls among distractors, presented on a 3D television or in a VR headset. The number of targets and speed of balls adaptively increases with practice.
Nintendo Brain Age (http://brainage.nintendo.com)	General population.	The first product to bring “brain training” to a mass market. based largely on a 2003 book of puzzles and exercises by neuroscientist Ryuta Kawashima.	WM capacity and associated functions (e.g., concentration, focus).	Available in “App” format and its traditional console-based platform. Brain Age uses mini-games that require players to complete math problems quickly, read aloud, or perform other spatial, verbal, and arithmetic tasks.
Dynavision (www.dynavisioninternational.com/)	Medical, athletic and military.	Designed to improve visuo-motor as well as cognitive skills. The product is marketed as a training apparatus and as a tool for concussion diagnosis, rehabilitation, and return to play decisions.	Vision, cognition, motor control, concentration, decision-making.	Wall-mounted, computer-driven light board fitted with 70 lit buttons. Requires users to recurrently tap the buttons, when lit, as quickly as possible.

**Table 3A:** Summary of compensatory and restorative studies (older adults and populations with health conditions).

**Article**	**Sample (completed)**	**Device**	**Active Control**	**Near transfer test**	Far transfer^*^ test	**Retention test**	**Independent of company**	**Cognitive outcomes**	**Findings**	
Ackerman et al., 2010	78 healthly adults (mean 60.7 yrs)	Wii Big Brain Academy	Reading exercises	Yes	No	No	Yes	Fluid and crystalline intelligence, processing speed	There was an effect of training on measures of processing speed (*ps* < 0.01) and fluid intelligence (verbal tests) (*ps* < 0.01), but there was no benefit for the TG compared to the control group for any measures.	
Åkerlund et al., 2013	47 participants with impaired WM following traumatic brain injury (47.7 ± 11.3 yrs)	Cogmed	No	Yes	Yes (SR)	No	Yes	STM, WM	Both TG and control improved on digit span (STM). The TG showed a significantly greater improvement in digit span (*p* = 0.045). TG did not show greater improvement on other measures of WM (spatial span, sequence memory). There was no self-reported change in executive function or psychological health for either group.	
Ballesteros et al., 2014	30 healthy older adults (57–80 yrs)	Lumosity	No	Yes	Yes (SR)	No	Yes	Processing speed, selective attention, EF, spatial working memory, episodic memory	TG showed greater improvement than controls in oddball task performance (selective attention), speed of processing and Wechsler memory (*ps* < 0.05), but not EF, spatial WM or self-reported wellbeing.	
Björkdahl et al., 2013	45 adults with WM deficits following brain injury (51.0 ± 11 yrs)	Cogmed	No	Yes	Yes	Yes	Yes	STM	TG showed significant improvement in STM (digit span) following training (*p* = 0.003), with no change in the control group. Both groups improved on therapist ratings of motor skill.	
Brehmer et al., 2011	23 healthy older adults (mean 63.7 yrs)	Cogmed	Non-adaptive version of training task	Yes	No	No	No	WM, sustained attention, inhibition, STM reasoning	Interaction effects indicated greater gains in divided attention and WM (span tasks) for the TG (*p* < 0.05). This was not found for inhibition, STM and reasoning. Training gains were related to changes in neural activation.	
Charvet et al., 2015	20 participants with cognitive impairment due to MS. (39.8 ± 11.5 yrs)	Lumosity	Computer games	Yes	Yes	No	Yes	Memory, processing speed	There was a significant difference between TG and active controls post-training in composite cognitive score (*p* = 0.02) but not for individual tests (e.g., WAIS, Corsi blocks). TG also performed better on a motor function task (*p* = 0.01).	
Edwards et al., 2013a	74 adults (>40 yrs) with Parkinson's disease (68.9 ± 8.1 yrs)	InSight, Posit Science	No	Yes	Yes (SR)	No	No	Visual speed of processing	TG showed significantly greater improvements in visual processing speed (useful field of view) (*p* = 0.032). TG did not differ from controls in self-reported cognitive performance or depressive symptoms.	
Edwards et al., 2013b	67 healthy older adults (74.0 ± 7.5 yrs)	InSight, Post Science	No	Yes	Yes (SR)	No	No	Visual speed of processing	TG showed significantly greater improvements in visual processing speed (useful field of view) (*p* = 0.043) than wait-list controls. There was no effect of training on self-reported social or cognitive function.	
Finn and McDonald, 2011	16 older adults with mild cognitive impairment (72.7 ± 7.1 yrs)	Lumosity	No	Yes	Yes (SR)	No	Yes	Sustained attention, WM, set shifting, visual memory	TG showed greater improvement on sustained attention, but not WM, memory (pattern recognition) or shifting (set shifting). Also no effect on subsequent training of waitlist controls. No effect on self-report of mood.	
Gropper et al., 2014	62 university students with ADHD or learning disabilities (28.0 ± 7.2 yrs)	Cogmed	No	Yes	Yes (SR)	Yes	Yes	WM, sustained attention, selective attention, reading and mathematics comprehension	There was no effect of training on WM (digit span), sustained or selective attention or mathematics and reading comprehension (*ps* > 0.05). There were similarly no group differences at 2-month follow-up. There was a reduction in self-reported ADHD symptoms.	
Haimov and Shatil, 2013	51 older adults with insomnia (65–85 yrs)	Cognifit	Simple computer tasks	Yes	Yes	No	No	Range of tests including: memory, divided attention, inhibition, shifting, WM, processing speed	TG showed improvements in several functions, including memory (*p* < 0.001), divided attention (*p* < 0.05), processing speed (*p* < 0.01), visual WM (*p* < 0.001). The TG showed greater improvements than the active control in memory (*p* < 0.001), visual (*p* < 0.001), and auditory (*p* < 0.001) WM. TG also showed improvements in sleep quality.	
Hellgren et al., 2015	48 adults with acquired brain injury (mean 43.7 yrs)	Cogmed	No	Yes	No	No	Yes	WM, processing speed, sustained attention, divided attention	The TG improved on all tests of WM and attention (*ps* < 0.001). TG reported increased quality of life (*p* < 0.001). No control group comparison.	
Hyer et al., 2016	68 older adults (>65 yrs) with memory impairment	Cogmed	Non-adaptive version of training task	Yes	No	Yes	Yes	WM, executive function	The TG showed greater improvements than active controls on one of two WM span tests (*p* = 0.01), but not on an executive function test.	
Kesler et al., 2013	41 women with history of breast cancer (56.0 ± 7.0 years).	Lumosity	No	Yes	No	No	Yes	Executive function, WM, processing speed	The TG showed significantly greater improvement than controls in EF (WCST) (*p* = 0.008) and processing speed (symbol search) (*p* = 0.009) but not WM (digit span) (*p* = 0.57).	
Klavora et al., 1995	10 participants (45–80 yrs) unsafe to drive following stroke	Dynavision	No	Yes	Yes	Yes	Yes	Processing speed	There was a significant improvement in performance on the trained task (*ps* < 0.05) and an increase in the proportion of participants rated as safe to drive.	
Legault and Faubert, 2012	41 healthy older adults (64–73 yrs, mean 66.3)	Neurotracker	Perceptual task (contrast detection)	No	Yes	No	No	None	At a distance of 4 m the TG showed significantly better perception of partially masked human motion than active controls (*p* = 0.040). There was no difference at 16 m.	
Leung et al., 2015	209 healthy older adults (70.1 ± 6.4 yrs)	Brain Fitness, Posit Science	Educational programme	Yes	No	No	Yes	Sustained attention, WM, verbal STM,	The TG showed greater improvement on one of two sustained attention tests (*p* = 0.026) and on working memory (*p* = 0.012) but not STM.	
Liu et al., 2016 (study 2)	102 adults with ADHD (18–35 yrs)	Cogmed	No	Yes	No	No	Yes	WM, general intelligence	No effect of training group on changes in WM (delayed match to sample test) (*ps* > 0.05).	
Liu et al., 2017	88 young adults with ADHD (23.7 ± 3.3 yrs)	Cogmed	No	Yes	No	No	Yes	WM, fluid intelligence	No transfer to response control in Go/NoGo task.	
Lundqvist et al., 2010	21 adults with acquired brain injury (43.3 ± 9.8 yrs)	Cogmed	No	Yes	Yes (SR)	Yes	Yes	Divided attention, inhibition, switching, WM.	TG showed significant improvements in measures of WM, inhibition, switching, and divided attention immediately post-training (*p* < 0.001 to *p* = 0.002) and at 20-week follow-up (*p* < 0.001 to *p* = 0.002). There was no change in passive control group. TG also improved self-ratings of occupational performance.	
Mawjee et al., 2015	97 young adults (18–35 yrs) with ADHD (23.9 ± 3.4 yrs)	Cogmed	No	Yes	Yes (SR)	No	Yes	WM, STM, processing speed	There were no differences between TG and controls following training. There was also no difference in self-reported ADHD symptoms and cognitive failures.	
Mayas et al., 2014	27 healthy adults (57–77)	Lumosity	No	Yes	No	No	Yes	Alertness and distractibility	There was no effect of group (TG v control) on digit categorization performance in the oddball task. The TG significantly improved from pre to post in distractibility (*p* = 0.05) and alertness (*p* = 0.04).	
McDougall and House, 2012	41 healthy older adults (74.6 ± 8.5)	Nintendo Brain Age	No	Yes	No	No	Yes	Intelligence	Sub-tests of the WAIS only showed a benefit for backward digit span (*p* < .05). There was no effect for vocabulary, block design, arithmetic and forward digit span. There was no effect of more frequent use.	
Nouchi et al. (2012)	28 healthy older adults (69.1 ± 2.4 yrs)	Nintendo Brain Age	Video game	Yes	No	No	Yes	Executive function, WM, processing speed	Following training, the TG showed significantly greater improvements in EF (*ps* = 0.001–0.006) and processing speed (*ps* = 0.005–0.014). There was no difference in WM (digit span) improvement between groups (*p* > 0.05)	
Peretz et al., 2011	155 healthy older adults (68 ± 7 yrs)	Cognifit	Video games	Yes	No	No	No	Overall cognitive performance	There was a significant improvement in overall cognitive score in the TG (*p* < 0.05) and the active control (*p* < 0.05). There was no difference in improvement between groups.	
Preiss et al., 2013	31 participants with unipolar and bipolar depression (44.2 ± 14.2 yrs)	Cognifit	No	Yes	Yes (SR)	No	No	WM, shifting, inhibition, divided attention, STM, executive function	There was no difference between TG and controls for WM, shifting, inhibition, divided attention, STM, or executive function (Stroop, WCST). Improvements in self-report of depressive symptoms.	
Rass et al., 2015	56 methadone maintenance patients (43.4 ± 8.0 yrs)	Cogmed	Non-adaptive version of training task	Yes	Yes (SR)	No	Yes	WM, STM, processing speed, reasoning, inhibition	Greater WM (digit span, OSPAN) improvements in the TG than controls (*p* = 0.003). No group differences in improvement in processing speed (trail making), inhibition or reasoning. TG reported less drug use post-training than active controls (*p* = 0.045).	
Siberski et al., 2015	32 adults with intellectual and developmental disabilities (40.5 ± 11.0 yrs)	Cognifit	Video games	Yes	No	No	No	Divided attention, inhibition, shifting, processing speed, WM	The TG improved in measures of monitoring (*p* = 0.017), visual WM (*p* = 0.003), and processing speed (*p* = 0.038) but not divided attention, shifting (WCST), or inhibition (Stroop). There were no group differences for any measure post-intervention.	
Smith et al., 2009	487 healthy older adults (>65 yrs)	Brain Fitness, Posit Science	Educational training	Yes	No	No	No	Cognitive assessment battery (inc. attention and memory), WM	The TG showed greater improvement than controls in the cognitive battery (*p* = 0.02) and WM task (*p* = 0.006).	
Strenziok et al., 2014	42 healthy older adults	Brain Fitness, Posit Science	Video games	Yes	No	No	Yes	Reasoning, WM, STM.	The TG showed a significant improvement in reasoning (WAIS matrix) scores (*p* < 0.05) but no improvement in WM (letter number sequencing).	
Von Ah et al., 2012	82 breast cancer survivors (56.5 ± 8.5 yrs)	InSight, Posit Science	Memory training	Yes	Yes (SR)	Yes	No	Memory, speed of processing	TG showed enhanced processing speed (useful field of view test) in comparison to passive controls post-training (*p* = 0.040) and at 2-month follow-up (*p* = 0.016). Also improved memory post-training (*p* = 0.0004) and at follow-up (*p* = 0.010). TG improved self-reported cognitive functioning (*p* = 0.042).	
Wentink et al., 2016	107 adults (45–75 years) recovering from stroke	Lumosity	No	Yes	Yes (SR)	Yes	Yes	WM, inhibition, fluid intelligence	TG outperformed controls in one of four WM tests (*p* = 0.02) and an inhibition test (*p* < 0.001) post-training. At 16-week follow-up there were no group differences in WM, inhibition, attention, and fluid intelligence tests. Also no differences in self-reported cognitive failures or quality of life.	

* *Transfer to tasks other than laboratory cognitive tests*.

*SR, self-report outcome; WM, working memory; STM, short-term memory; EF, executive function; TG, treatment group; OSPAN, Operation Span task; WCST, Wisconsin card Sorting Task; WAIS, Wechsler Adult Intelligence Scale; ADHD, Attention deficit hyperactivity disorder; MS, Multiple Sclerosis; ps, multiple p-values*.

Quality assessment color key: Strong (80+%)Fairly strong (70–79%)Moderate (60–69%)Weak  (50–59-%)Very weak (460–49%)

**Table 3B:** Summary of studies using healthy and elite populations (additive studies).

**Article**	**Sample (completed)**	**Device**	**Active Control**	**Near transfer test**	Far transfer^*^ test	**Retention test**	**Independent of company**	**Cognitive outcomes**	**Findings**	
Brehmer et al., 2012	55 younger adults (mean 26.0 yrs) and 45 older adults (mean 63.8 yrs)	Cogmed	Non-adaptive version of training task	Yes	No	Yes	No	WM, sustained attention, inhibition, STM, reasoning	TG showed greater improvement in WM tasks (forwards and backwards span) than controls (*p* = 0.01 and *p* < 0.001). TG also showed greater improvement in sustained attention, but not inhibition, STM or reasoning. Group differences remained at 3 month follow-up	
Dunning and Holmes, 2014	45 students (18–21 yrs)	Cogmed	Non-adaptive version of training task	Yes	No	No	Yes	WM, STM	Interaction effects indicated greater gains on verbal and visuospatial WM (span tasks) and verbal STM for the TG over active and passive controls (*ps* < 0.05), but not for visuospatial STM.	
Gibson et al., 2013	20 undergraduate students	Cogmed	No	Yes	No	No	Yes	WM	Two TGs displayed significantly greater recall on items from primary (*p* < 0.05) and secondary memory (*p* < 0.01) than passive controls.	
Hardy et al., 2011	23 participants (mean 57.0 yrs)	Lumosity	No	Yes	No	No	No	Spatial WM and divided visual attention	TG improved significantly from pre to post in divided attention (*p* < 0.001) and significantly more than controls (*p* = 0.027). TG improved significantly in forward spatial WM, (*p* = 0.032), significantly more than controls. They also improved reverse spatial span (*p* = 0.008). There was no change in letter memory, (*p* = 0.517).	
Hardy et al., 2015	4715 Lumosity users (18–80 years; 39.2 ± 15.1 yrs)	Lumosity	Crossword Puzzles	Yes	Yes (SR)	No	No	Overall battery (inc. STM, WM, grammatical and arithmetic reasoning, response inhibition, selective attention)	Significantly greater improvement on battery in TG (*p* < 0.001). Largest effects in inhibition and arithmetic reasoning. Also significantly greater improvement in self-reported cognition and emotional status, (*p* < 0.001).	
McNab et al., 2009	13 healthy males (20–28 yrs)	Cogmed	No	Yes	No	No	No	WM	Training improved WM capacity (*p* < 0.001). No comparison group. Improvements were associated with cortical dopamine binding.	
Metzler-Baddeley et al., 2016	40 adults (26.5 ± 6.6 yrs)	Cogmed	Non-adaptive version of training task	Yes	Yes	No	Yes	WM, inhibition, grammatical reasoning, general intelligence, multi-tasking	The TG showed significantly greater improvement in two measures of WM (*ps* < 0.001), but not in tests of inhibition, grammatical reasoning, general intelligence, and multi-tasking. Adaptive training related to structural brain changes measured through Magnetic Resonance Imaging.	
Nouchi et al., 2013	32 young adults (20.7 ± 1.2 yrs)	Nintendo Brain Age	Video game	Yes	No	No	Yes	Fluid intelligence, EF, WM, STM, processing speed	The TG showed greater improvements than active controls in EF (*ps* < 0.001–0.002), WM (OSPAN) (*p*s = 0.003–0.008) and processing speed (symbol search) (*ps* = 0.004–0.006). Active controls showed greater improvement in sustained attention (*p* = 0.01) and visuo-spatial ability (*p* = 0.009). No improvement in fluid intelligence or STM for either group.	
Parsons et al., 2016	20 University students (23.3 ± 2.7 yrs)	Neurotracker	No	Yes	Yes	No	No	Selective and sustained attention, processing speed, STM, WM, inhibition	TG showed significant improvements in sustained attention (*p* = 0.007), inhibition (*p* = 0.004), WM (*p* = 0.02), and STM (*p* = 0.008) (WAIS tests). TG also showed decreased EEG power in theta, alpha and delta bands, primarily in frontal cortex.	
Romeas et al., 2016	23 soccer players (21.7 ± 0.5 yrs)	Neurotracker	Soccer videos	No	Yes	No	No	None	TG showed significantly greater improvement than controls in passing accuracy (*p* = 0.044), but not dribbling or shooting. There was a significant increase in self-reported confidence in decision making in the TG (*p* = 0.012) but not controls.	
Vartanian et al., 2016	41 Armed Forces personnel (21–50 years)	Neurotracker	Dual n-back	Yes	No	No	Yes	WM	TG showed significant increases in word (*p* = 0.005), visual (*p* = 0.05), and matrix (*p* = 0.015) span tasks. There was no improvement in active and passive control groups.	

* *Transfer to tasks other than laboratory cognitive tests*.

*SR, self-report outcome; WM, working memory; STM, short-term memory; EF, executive function; TG, treatment group; OSPAN, Operation Span task; WCST, Wisconsin card Sorting Task; WAIS, Wechsler Adult Intelligence Scale; ADHD, Attention deficit hyperactivity disorder, MS, Multiple Sclerosis; ps, multiple p-values*.

Quality assessment color key: Strong (80+%)Fairly strong (70–79%)Moderate  (60–69%)Weak  (50–59-%)Very weak (460–49%)

#### Cogmed

Cogmed was found to have the most extensive research base with 15 studies matching the criteria, many of which recruited populations with cognitive impairments. Several showed good evidence for near transfer effects, for instance, Åkerlund et al. (2013), Björkdahl et al. (2013), and Dunning and Holmes (2014) all found greater improvement on working memory tasks in the training group than controls. There were, however, null findings regarding working memory improvements in the studies of Gropper et al. (2014), Liu et al. (2016, 2017), and Mawjee et al. (2015). Additionally there were few findings showing improvements in related areas, such as executive function (but cf. Hellgren et al., 2015). With regards to testing far transfer, the Cogmed studies used almost exclusively self-report outcomes, such as quality of life and health condition symptoms. The one exception was Metzler-Baddeley et al. (2016) who found changes in cortical thickness as a result of training. Some of the Cogmed studies provided the best examples of an active control group (Brehmer et al., 2012; Dunning and Holmes, 2014; Metzler-Baddeley et al., 2016), with participants given the same (but non-adaptive) tasks as the trainees. Additionally, two studies assessed skill retention (Brehmer et al., 2012; Gropper et al., 2014) and several of the positive findings came from independent research groups.

Overall, there is good evidence, albeit with some null findings, for near transfer effects following Cogmed training. Some studies also found this to extend to self-rated improvements in everyday life, but there were no studies extending the observed working memory benefits to tasks representative of daily life or sporting activities.

#### Lumosity

Like Cogmed, several of the nine included Lumosity studies used populations with health conditions (Finn and McDonald, 2011; Charvet et al., 2015; Wentink et al., 2016), but the device has also been tested in healthy populations more relevant to sport. In particular, a large trial of 4,715 participants ranging from 18 to 80 years (Hardy et al., 2015) provides a more generalizable sample. In this study, the training group showed greater improvements than active controls (crossword puzzles) in a range of cognitive tests assessing working memory, executive function, and attention. Across the studies there was good support for the benefits of Lumosity training for near transfer in several cognitive functions, such as speed of processing (Ballesteros et al., 2014), working memory (Hardy et al., 2011), and executive function (Kesler et al., 2013). There were also some null findings for near transfer, but in a small sample (Finn and McDonald, 2011). Regarding far transfer effects, Charvet et al. (2015) found improved motor skill in multiple sclerosis patients, but other studies found no change in self-reported wellbeing (Ballesteros et al., 2014) or mood (Finn and McDonald, 2011).

The study of Hardy et al. (2015) provided easily the largest cohort of the studies in this review, but as participants were already Lumosity users, who were compensated with Lumosity membership, these findings should be viewed with caution. Nevertheless, many of the findings were independent of those manufacturing the product. Overall, despite support for near transfer, there was no evidence of retention, and there has been limited assessment of real-world transfer or additive benefits, the key criteria for generalizing to sport. Findings of improved motor function suggest potential benefits, however this was observed in a population living with multiple sclerosis. Overall there is little evidence that Lumosity training can transfer to tasks beyond the lab.

#### Posit science

All six studies meeting the review criteria reported positive effects of Posit Science training for near transfer, mainly in older adults. Improvements in processing speed (Edwards et al., 2013a,b), working memory (Smith et al., 2009; Leung et al., 2015) and short-term memory (Von Ah et al., 2012) were found, predominantly in older adults. Tests of transfer were confined to self-report measures with no real-world tasks relevant to sport, and only weak benefits were found. While Von Ah et al. (2012) found a marginal benefit for perceived cognitive function, Edwards et al. (2013a,b) observed null effects. Several studies used active controls, such as educational training (Smith et al., 2009; Leung et al., 2015), and positive findings have been replicated by independent researchers (Strenziok et al., 2014; Leung et al., 2015). As was the case for most devices, no retention of skills was assessed. Overall, studies supported near transfer effects for compensatory/restorative training, but no evidence for additive effects.

#### Cognifit

Four studies were identified that directly assessed Cognifit training, across healthy older adults, adults living with intellectual disabilities, adults living with insomnia sufferers and participants living with depression. Three of the four studies found evidence for near transfer benefits (Peretz et al., 2011; Haimov and Shatil, 2013; Siberski et al., 2015), but there were several null effects across these studies and one other showing exclusively null effects (Preiss et al., 2013). The only test of far transfer was self-rated improvement in depressive symptoms (Preiss et al., 2013), which did indicate benefits. These studies generally used appropriate active control groups, principally other computer games (Peretz et al., 2011). Unfortunately there was no test of retention and all studies were conducted by researchers with ties to Cognifit. Overall, the evidence for near transfer effects was relatively weak, and there was no evidence of transfer to tasks representative of sport.

#### Neurotracker

Four studies investigating Neurotracker were included in the review, although the website lists further studies indicating that Neurotracker ability correlates with sporting (Faubert, 2013; Mangine et al., 2014), driving (Michaels et al., 2017), and surgical (Harenberg et al., 2016) performance. The research base for Neurotracker differs somewhat from those of Cogmed, Lumosity, and Posit Science, which have focused almost exclusively on near transfer effects. Only two of the Neurotracker studies actually tested near transfer effects; Parsons et al. (2016) found improvements in sustained attention, inhibition and working memory following training, while Vartanian et al. (2016) similarly found improvements in several measures of working memory. There is, conversely, more evidence for far transfer effects, and greater use of young and healthy populations, in comparison to other devices.

Firstly, Parsons et al. (2016) found training effects to be accompanied by changes in resting state brain function, primarily decreased theta, alpha, and delta EEG bands in the frontal cortex following 10 training sessions. Secondly, among older adults with impairments in perceiving biological motion, Legault and Faubert (2012) found significant improvements in identifying point light walkers (coordinated moving dots that simulate human motion) at a distance relevant for collision avoidance. Of most relevance for current purposes, is a study by Romeas et al. (2016) which provided the only study in this review to directly test transfer to a sporting task. Romeas et al. (2016) found significant improvements in coach ratings of passing accuracy following Neurotracker training, however, the small sample size (<10 per group) and the null effects for dribbling and shooting should, however, be taken into account. Three of the four studies used appropriate active controls, such as a working memory task, but there was no testing of retention.

Overall, the evidence for far transfer effects and sporting benefits in particular is more promising than most devices. Transfer effects have been found for perception of motion and soccer passing, with EEG suggesting measurable changes in neural activity. Nonetheless the evidence for near transfer is weaker than other devices, and studies have, for the most part, used small samples and been conducted by researchers connected to the company.

#### Nintendo's brain age

Four studies included in the review assessed Nintendo's Brain Age and Big Brain Academy, which provided mixed findings for near transfer effects. Two studies, conducted by Nintendo's researchers, found improvements in executive function, processing speed, and working memory following training, relative to computer game controls (Nouchi et al., 2012, 2013). Conversely, Ackerman et al. (2010) found no benefit to the training group above controls, and McDougall and House (2012) found null effects across most sub-measures of the Wechsler Adult Intelligence Scale. Therefore the evidence for even near transfer effects is weak. Additionally, there are no studies testing far transfer effects of Nintendo's products or retention of abilities. Hence, there was little support for this device and no evidence for sporting transfer.

#### Dynavision

One study, conducted by independent researchers, was identified that employed a Dynavision training intervention. There is currently little evidence regarding the cognitive functions that are directly targeted by Dynavision as the one included paper inferred improvements in processing speed from the trained task, and did not employ other cognitive measures (Klavora et al., 1995), so there is no evidence of near transfer. There is, however, initial evidence for far transfer, as Klavora et al. (1995) found 10 participants assessed as unsafe to drive following a stroke, to show significantly improved driving ability following training. Unfortunately, this study did not use an active control group, or assess retention of the improvement in driving. Overall the evidence base for this device is weak, as even near transfer to other cognitive tasks is yet to be established and there has been no test of sporting transfer.

### Quality assessment

Scores ranged from 40.9 to 81.8%, with a mean of 62.2% (Appendix 1 in Supplementary Material). Overall, studies scored highly in items relating to the tasks used, basic design, making clear hypotheses, reporting the main findings, assessment tasks, and measuring near transfer. The lowest scoring item was inclusion of a transfer task representative of real-world performance, which was only achieved in four studies. Additionally, only seven studies included justification of sample size, and only eight assessed retention of trained skills. Other issues that were poorly addressed were consistent reporting of effect sizes and the generalizability of findings, due to many studies using niche or non-healthy populations. Eighteen of the 43 studies were carried out by researchers with known connections to the companies.

## Discussion

The aim of this systematic review was firstly to evaluate the evidence that currently exists for the effectiveness of CCT devices, and secondly the evidence for transfer to sporting performance. In principle, regular training of key cognitive abilities may hold great value for sporting scenarios, which place high demand on attentional and processing resources, requiring decisions to be made under pressure (Ducrocq et al., 2016). Currently, however, there is a gulf between scientific findings and marketing claims. Therefore, we aimed to provide a rigorous overview of the peer-reviewed evidence for these devices. With regards to our stated aims, the CCT devices showed limited evidence for far transfer effects in general, and evidence of additive effects relevant to sport was particularly scarce, mainly because only one study directly assessed transfer to a sporting task.

### Summary of evidence

The premise of CT is that training of core cognitive abilities will transfer to other tasks and environments. As such, while there was good evidence for near transfer effects in many devices (as has been found in other reviews; Melby-Lervåg and Hulme, 2013), this is not sufficient to conclude overall device effectiveness. Within the compensatory/restorative studies there was limited evidence for far transfer effects beyond the trained tasks, and where transfer tests were used, they often consisted of self-reporting of symptoms. This is a particular problem given the sporadic use of active control groups. Overall, evidence is currently weak for real world benefits from CCT devices, even in deficit populations where we might expect the largest effects. With regards to the narrower focus on potential sporting benefits, the evidence reviewed provides little indication that CCT devices can transfer to the sports field. Firstly, the number of studies using tasks and populations that can be generalized to sport was almost null, with only one study directly using a sporting transfer task. Secondly, the lack of transfer across all populations is not encouraging for athletes who are seeking additive effects. The underwhelming quality of the studies assessed means that positive effects cannot yet be ruled out, but there is little current evidence for them.

Based on the results of the review, the findings relating to Cogmed, Lumosity, Cognifit, and Posit Science^4^ could be grouped together due to similarity of training method and published evidence. These devices use online or app-based games, which closely mimic traditional cognitive tasks, such as memory span and dual load tasks. Their evidence base for near transfer effects is fairly strong, and these devices likely enhance working memory, processing speed, executive function, and attention in laboratory based tasks (Melby-Lervåg and Hulme, 2013). There was, however, very little testing of far transfer effects or retention of trained skills. Whether far transfer tasks have been employed, but remain in the “file drawer” due to null effects, cannot be known. Therefore, these devices hold little promise for benefiting sporting performance.

Outside of this group, Neurotracker provided a training option that included a greater perceptual element and aimed to be more representative of sporting skills. In comparison to other devices, there was relatively little direct testing of near transfer effects, but findings are rather more promising for transfer to real-world tasks. Studies provided initial evidence for enhancing human motion perception (Legault and Faubert, 2012) and soccer passing (Romeas et al., 2016); an indication of far transfer that was absent from the first group of devices. Studies with this device are yet to assess retention effects, following a period without device use. As such further study is required to understand whether beneficial effects rely on persistent use, or can be achieved from a single intervention. In addition, Dynavision training, which similarly included a perceptual element, has been linked to improvements in driving ability (Klavora et al., 1995), but here the evidence was relatively weak. Consequently, while these findings certainly warrant further consideration, firm conclusions cannot yet be drawn as these studies suffer from the same methodological issues discussed previously. In summary, adopting any of the reviewed devices for training athletes would be based on a belief in the principles of domain generality and neuroplasticity rather any conclusive evidence of transfer effects. While these devices may benefit performance in similar, laboratory-based tasks, there is currently weak evidence of their value for sport.

### Quality assessment

Quality assessment scores (Appendix 1 in Supplementary Material) suggest that, overall, the studies in this area display several methodological issues. Some particular concerns include basic experimental design issues like calculation of sample size. A number of the papers reviewed (13) had small samples (<15 per group) with no power analysis as justification. As a result, many of the studies in this area are likely underpowered, meaning the positive findings that do exist have an increased chance of being erroneous (Button et al., 2013). Additionally, many studies included batteries of cognitive tests, which created a multiple testing issue that was, in general, ignored. Preregistration^5^ of planned analyses would be a major step forward in avoiding an ad hoc approach to assessing training effects in this area (Simons et al., 2016).

Methodological choices of the included studies have also limited the conclusions that can be drawn about transfer to sport. In particular the lack of representative real-world tasks and assessment of retention mean that extending findings to sporting scenarios is problematic. Similarly, participant populations often had cognitive deficits, limiting generalizability to healthy populations, where effect sizes may well be smaller. For CCT devices to provide convincing evidence for sporting benefits, these questions must be addressed in future studies.

### Future directions

Future work in this area should focus on the devices that hold the greatest promise for sporting transfer, namely those with a perceptual-cognitive element, more representative of the demands of sport. More studies are required that use athlete populations (rather than cognitively impaired) and test transfer to more representative tasks. Studies must, however, take note of the methodological issues that are prevalent in this area (Simons et al., 2016). As this literature is particularly prone to selective reporting of tests and results, preregistration of accurately powered trials is imperative. The use of adequate active control groups must also be improved, to allow a fair comparison of training effects. CT is an area where much research to date could claim to be “exploratory,” but in order to move toward any kind of reliable evidence, a more systematic approach, which rectifies many of the methodological issues, is required.

### Limitations

As with any systematic review, the conclusions must be taken within the context of the search criteria. Other methods of training cognitive function are available, such as transcranial direct stimulation, mindfulness, and exercise interventions. Additionally, amalgamations of interventions were not included, hence the efficacy of combined training strategies cannot be ruled out. There are also a large number of excluded studies which use non-commercially available devices. These studies may report more convincing methods or effects, indeed much working memory training research is more rigorous (see Melby-Lervåg and Hulme, 2013; Ducrocq et al., 2016). We suggest, however, that a focus on commercial devices was warranted given their growing popularity, easy access, endorsements, and the confusion about their effectiveness in the sporting community.

## Conclusions

In this systematic review we aimed to evaluate the evidence currently available for CCT devices. Through assessing study quality and synthesizing the available results, it is apparent that there is limited evidence that improvements found in lab-based cognitive tasks transfer to real world benefits. In particular, the very limited use of populations and tasks representative of sport means inferences about CCT effectiveness for athletes are unreliable. Additionally, we identified a series of methodological issues within the CCT literature, such as use of appropriate controls, small sample sizes, lack of retention tests and limited replication of findings by independent researchers. Companies promoting CCT products must address these issues in order to make scientifically valid claims about device effectiveness, while those in the sporting community looking to adopt the use of these products should seek to verify device claims with a healthy degree of skepticism.

## Author contributions

All authors contributed to the review design, search criteria, and writing of the paper. DH and SV conducted the paper search and assessment.

### Conflict of interest statement

The authors declare that the research was conducted in the absence of any commercial or financial relationships that could be construed as a potential conflict of interest.

## Footnotes

^1^Older adult samples were generally classified as compensatory/restorative when participants were over 60 years of age, but this was somewhat guided by the intentions of individual studies.

^2^We have classified far transfer as any test that was not a laboratory cognitive test. In several cases this included self-report of daily functioning and symptoms of health conditions. Any self-report tests are identified in the results.

^3^Some papers used “far transfer” to refer to enhancement of a cognitive function that was not directly trained, but here, due to the overlapping nature of concepts like working memory, executive function, and fluid intelligence, we restrict the term to real-world tasks or benefits.

^4^Nintendo's device could also be included here, but its evidence base is somewhat weaker.

^5^Recording intended methods and analyses prior to data collection.
