# Supplementary material for: A Systematic Review of Commercial Cognitive Training Devices: Implications for Use in Sport
Source: Front Psychol. 2018 May 11;9:709. doi: 10.3389/fpsyg.2018.00709 (PMC5958310; doi:10.3389/fpsyg.2018.00709)
Supplement: Supplementary file 1 [file Data_Sheet_1.PDF]

Appendix 1; Quality assessment scores

Article	Items																						Total	
	1	2	3	4	5	6	7	8	9	10	11	12	13	14	15	16	17	18	19	20	21	22	Raw	%
Ackerman, Kanfer & Calderwood (2010)	1	0	1	1	0	1	1	1	1	1	1	0	1	0	0	1	1	1	0	1	0	1	15	68.2
Akerlund, Esbjörnsson, Sunnerhagen & Björkdahl (2013)	1	1	1	1	1	1	1	1	0	1	1	0	1	0	0	0	1	1	0	0	0	1	14	63.6
Ballesteros, Prieto, Mayas, Toril, Pita, Ponce de Leon, Reales & Waterworth (2014)	1	1	1	1	1	1	1	1	1	1	1	0	1	0	1	1	1	1	0	0	0	1	17	77.3
Björkdahl, Åkerlund, Svensson & Esbjörnsson (2013)	0	1	1	1	1	0	1	0	0	1	1	0	0	1	0	0	1	1	0	0	1	1	12	54.5
Brehmer, Rieckmann, Bellander, Westerberg, Fischer & Bäckman (2011)	1	0	1	0	1	1	1	1	1	1	0	0	1	1	1	1	1	1	0	1	0	0	15	68.2
Brehmer, Westerberg & Bäckman (2012)	1	1	1	1	0	0	1	1	1	1	1	0	1	1	1	1	1	1	0	1	1	0	17	77.3
Charvet, Shaw, Haider, Melville & Krupp (2015)	0	0	1	1	1	0	0	1	0	1	0	0	1	0	0	0	1	1	1	1	0	1	11	50.0
Dunning & Holmes (2014)	1	1	1	1	1	1	1	1	1	1	0	0	0	1	0	1	1	1	0	1	0	1	16	72.7
Edwards, Hauser, O'Connor, Valdés, Zesiewicz & Uc (2013)	1	1	1	1	0	1	0	1	0	1	0	1	1	0	0	0	1	1	0	0	0	0	11	50.0
Edwards, Valdés, Peronto, Castora-Binkley, Alwerdt, Andel & Lister (2013)	1	1	1	1	0	0	1	1	1	1	0	0	1	1	0	1	1	1	0	0	0	0	13	59.1
Finn & McDonald (2011)	1	1	1	1	0	1	1	1	0	1	1	0	1	0	0	1	1	1	0	0	0	1	14	63.6
Gibson, Gondoli, Kronenberger, Johnson, Steeger, Morrissey (2013)	1	1	1	0	0	1	1	1	1	1	0	0	1	1	0	1	1	1	0	0	0	1	14	63.6
Gropper, Gotlieb, Kronitz & Tannock (2014)	1	1	1	1	1	0	1	1	1	1	0	0	1	1	0	0	1	1	0	0	1	1	15	68.2
Haimov & Shatil (2013)	1	0	1	1	1	0	0	1	1	1	1	0	1	0	0	0	1	1	0	0	0	0	11	50.0
Hardy, Drescher, Sarkar, Kellett & Scanlon (2011)	1	1	1	0	0	1	1	1	0	1	1	0	1	1	0	0	1	1	0	0	0	0	12	54.5
Hardy, Nelson, Thomason, Sternberg, Katovich, Farzin & Scanlon (2015)	1	1	1	1	1	0	1	1	1	1	1	1	1	1	0	0	1	1	0	1	0	0	16	72.7
Hellgren, Samuelsson, Lundqvist, Börsbo (2015)	1	1	0	0	1	0	0	1	0	1	0	0	1	0	0	1	1	1	0	0	0	1	10	45.5
Hyer, Scott, Atkinson, Mullen, Lee, Johnson & McKenzie (2015)	1	1	1	1	0	0	1	1	1	1	0	0	1	0	0	0	1	1	0	1	1	0	13	59.1
Kesler, Hosseini, Heckler, Janelsins, Palesh, Mustian & Morrow (2013)	1	1	1	1	1	1	1	1	0	1	0	0	1	1	0	1	1	1	0	0	0	1	15	68.2
Klavora, Gaskovski, Martin, Forsyth, Heslegrave, Young & Quinn (1995)	1	1	0	1	0	1	0	1	1	1	0	0	1	0	0	1	1	0	1	0	1	1	13	59.1
Legault & Faubert (2012)	0	1	1	0	0	1	1	1	0	1	0	0	0	0	0	0	1	0	1	1	0	0	9	40.9
Leung, Tam, Chu, Kwok, Chan, Lam, Woo & Lee (2015)	1	1	1	1	1	1	1	1	1	1	0	0	1	0	0	0	1	1	0	1	0	1	15	68.2

Liu, Glizer, Tannock & Woltering (2016) (study 2)	1	0	1	1	1	0	0	1	0	1	1	0	1	0	0	1	1	1	0	0	0	1	12	54.5
Liu, Lishak, Tannock & Woltering (2017)	1	1	1	1	1	0	0	1	1	1	1	0	1	0	0	1	1	1	0	0	0	1	14	63.6
Lundqvist, Grundström, Samuelsson & Rönnberg (2010)	1	1	1	1	1	1	1	1	0	1	0	0	0	0	0	0	1	1	0	0	1	1	13	59.1
Mawjee, Woltering & Tannock (2015)	1	1	1	1	1	0	0	1	1	1	1	0	1	0	0	0	1	1	0	0	0	1	13	59.1
Mayas, Parmentier, Andres & Ballesteros (2014)	1	0	1	0	1	0	1	1	1	1	0	0	1	1	0	1	1	1	0	0	0	1	13	59.1
McDougall & House (2012)	1	1	1	1	0	1	1	1	1	1	0	0	1	0	0	1	1	1	0	0	0	1	14	63.6
McNab, Varrone, Farde, Jucaite, Bystritsky, Forssberg, & Klingberg (2009)	1	0	1	0	0	0	0	1	1	1	0	0	1	0	0	1	1	1	0	0	0	0	9	40.9
Metzler-Baddeley Caeyenberghs Foley & Jones (2016)	1	1	1	1	1	1	1	1	1	1	0	0	1	0	0	0	1	1	0	1	0	1	15	68.2
Nouchi, Taki, et al. (2012)	1	1	1	1	1	1	1	1	1	1	0	0	1	1	0	0	1	1	0	1	0	1	16	72.7
Nouchi, Taki, et al. (2013)	1	1	1	1	1	1	1	1	1	1	0	1	1	1	0	1	1	1	0	1	0	1	18	81.8
Parsons, Magill, Boucher, Zhang, Zogbo, Bérubé, Scheffer, Beauregard & Faubert (2016)	1	1	1	1	1	1	1	1	0	1	1	0	1	0	1	1	1	1	0	0	0	0	15	68.2
Peretz, Korczyn, Shatil, Aharonson, Birnboim & Giladi (2011)	1	0	1	0	1	1	1	1	0	1	0	1	0	0	1	1	1	1	0	1	0	0	13	59.1
Preiss, Shatil, Cermáková, Cimermanová & Ram (2013)	1	1	1	1	1	0	0	1	1	1	1	1	1	0	0	1	1	1	0	0	0	0	14	63.6
Rass, Schacht, Buckheit, Johnson, Strain & Mintzer, (2015)	1	1	1	1	0	0	1	1	1	1	1	0	0	0	0	1	1	1	0	1	0	1	14	63.6
Romeas, Guldner & Faubert (2016)	1	1	1	1	1	1	1	1	0	1	1	0	0	0	0	1	1	0	1	1	0	0	14	63.6
Siberski, Shatil, Siberski, Eckroth-Bucher, French, Horton, Loefflad & Rouse (2015)	1	0	1	1	0	0	0	1	1	1	1	0	1	0	0	0	1	1	0	1	0	0	11	50.0
Smith, Housen, Yaffe, Ruff, Kennison, Mahncke & Zelinski (2009)	1	1	1	1	1	0	0	1	1	1	0	1	1	1	0	0	1	1	0	1	0	0	14	63.6
Strenziok, Parasuraman, Clarke, Cisler, Thompson & Greenwood (2014)	1	1	1	0	1	1	0	0	0	1	0	0	0	0	0	1	1	1	0	1	0	1	11	50.0
Vartanian, Coady & Blackler (2016)	1	1	1	1	1	1	1	1	1	1	1	0	1	1	0	0	1	1	0	1	0	1	17	77.3
Von Ah, Carpenter et al. (2012)	1	1	1	1	1	0	0	1	0	1	0	1	1	1	0	1	1	1	0	1	1	0	15	68.2
Wentink, Berger, de Kloet, Meesters, Band, Wolterbeek, Goossens & Vliet Vlieland (2016)	1	1	1	1	1	0	0	1	0	1	1	0	1	0	1	1	1	1	0	0	1	1	15	68.2
Overall mean																							13.7	62.2

Note: 1 – yes; 0 – no/ unknown;
