# Supplementary material for: A Systematic Review of Commercial Cognitive Training Devices: Implications for Use in Sport
Source: Front Psychol. 2018 May 11;9:709. doi: 10.3389/fpsyg.2018.00709 (PMC5958310; doi:10.3389/fpsyg.2018.00709)
Supplement: Supplementary file 2 [file Data_Sheet_2.PDF]

Appendix 2: Quality assessment items

Item Number	Item
1	Is the hypothesis/aim/objective of the study clearly described?
2	Are the main outcomes to be measured clearly described?
3	Is the study design clearly described and appropriate to test the hypotheses?
4	Are the characteristics of participants in the study clearly described?
5[a]	Is there evidence of attention to ethical issues?
6	Are the training tasks clearly described?
7	Are the targeted functions clearly described?
8	Are the main findings of the study clearly described?
9	Does the study provide estimates of the statistical parameters (e.g. regression coefficients)?
10	Are conclusions substantiated by the data that are presented in the results?
11	Are the subjects asked to participate in the study representative of the entire population from which they were recruited?
12[b]	Are details of sample size determination included?
13	Were the statistical tests used to assess the main outcomes appropriate?
14[c]	Are effect sizes consistently reported?
15	Can the study results be applied to other relevant populations?
16	Are results adequately compared to previous studies and in relation to theoretical frameworks?
17	Are the methods of assessing the outcome variables valid?
18[b]	Has transfer of training to other laboratory tasks that measure the same cognitive function as the training been measured?
19[b]	Has transfer to relevant real-world tasks been tested?
20[b]	Has the training been evaluated using an active control group whose members have the same expectations of the training benefits as the members of the experimental group?
21[b]	Is retention of the trained skills assessed?
22[b]	Is the research group independent of those selling the product?

Note: Items were taken from (Durant, 1994), the Quality Index (Downs & Black, 1998) and the Epidemiological appraisal Instrument (Genaidy et al., 2007), unless otherwise specified.

[a] Additional item to verify attention to ethics (Spencer, Ritchie, Lewis, & Dillon, 2003)

[b] Additional items from Simons et al, (2016)
